# Acute psychotic episode inaugurating a primary hyperaldosteronism: a case report

**DOI:** 10.11604/pamj.2022.43.103.32944

**Published:** 2022-10-26

**Authors:** Wiame El Bouchalli, Kaouthar El Mir, Salah-Eddine El Jabiry, Mohammed Berrimi

**Affiliations:** 1 Psychiatric Hospital, Hospital University CHU Mohammed VI, Oujda, Morocco

**Keywords:** Acute psychotic episode, Conn’s adenoma, primary hyperaldosteronism, case report

## Abstract

Psychiatrist manifestations-especially acute psychotic episode-of primary hyperaldosteronism is not very common in the scientific literature. The main symptoms are high blood pressure and hypokalemia, the diagnosis seems to be based on the determination of aldosterone and plasma renin activity, also the treatment includes majorly adrenalectomy. The take-away lesson from this case is to think of organic cause in front of acute psychotic episode.

## Introduction

Primary hyperaldosteronism is a syndrome described by Conn in 1955, it is caused by an inappropriate autonomic secretion of aldosterone with suppression of the rate of renin. It is characterized by high blood pressure, associated or not with hypokalemia [1]. Clinical and biological manifestations boil down to polyuria and polydipsia, diastolic arterial hypertension, hypokalemia and hypernatremia, hypervolemia and hypokalemic alkalosis, producing episodes of asthenia, paresthesia, transient paralysis and tetany [2]. Conn syndrome may be due to Conn's adenoma (benign tumor of the adrenal corticosteroid), bilateral adrenal hyperplasia and exceptionally corticoadrenal carcinoma [3]. Hyperaldosteronism can be complicated by fibrosis that can affect several organs, including the heart. Cardiac involvement with a type of hypertrophic cardiomyopathy, even in the absence of high blood pressure [4]. The diagnostic approach is based on the determination of aldosterone and plasma renin activity, which is > 0.95 (in nmol/l) or > 35 (in ng/dl). A confirmatory test, with fludrocortisone is carried out afterwards, it is the most efficient. Imaging (CT or MRI) is required to determine if it is a tumor or adrenal hyperplasia, treatment includes adrenalectomy associated with aldosterone blockers such as spironolactone or eplerenone [3]. Psychiatric manifestations related to primary hyperaldosteronism have been described in the literature, including depression to anxiety disorders [5]. We report the case of an acute psychotic episode in a young patient aged 26 years who required a first hospitalization in psychiatry before the discovery of primary hyperaldosteronism following iterative hypertensive peaks in intra-hospital.

## Patient and observation

**Patient information:** this is MA, a 26-year-old, single, engineer with no medical or surgical history. He has a psychiatric history of attempting suicide 7 years ago by drug ingestion in a context of depressive episode following a school failure. The patient began using tobacco and cannabis at the age of 23, while alcohol consumption was occasional.

**Timeline:** the medical history goes back 9 days before his admission by the apparition of insomnia, unmotivated laughter, a hallucinatory behavior, persecutory ideation, complicated 4 days later, by the appearance of a physical aggressivity towards his mother. On the day of the admission, he was brought back by the police for disturbing public order (In a mosque, he tried to make the call to prayer in an anarchic way).

**Clinical findings:** the psychiatric assessment found a stable patient, anxious, his mood was slightly sad, he had a rich delusional symptomatology with strong emotional participation. The delusional themes were most likely religious and spiritual (I am the prophet Ahmed), persecutory (I am pursued, spied on), and so many reference's ideas (films tell my story, radio broadcasts talk about me) and black magic. The delusional mechanism was mostly intuitive with unwavering conviction. We did not note any hallucinatory symptoms or suicidal ideations. The somatic examination was without particularities.

**Diagnostic assessment:** the patient received a complete biological assessment returning normal, the diagnosis of an acute psychotic episode was retained, and then he was put on Aripiprazole at the dose of 10 mg per day combined with diazepam to control his anxiety. During his hospitalization, the patient had high blood pressure figures associated with sweating and paroxysmal palpitations. He had benefited from a blood ionogram that objectified a deep hypokalemia motivating his transfer to the intensive care unit where he was hospitalized for a day before being transferred to the cardiology department or he benefited from a thyroid assessment and kidney function returning without particularities. A chest abdomen and pelvis scan was performed, objectifying a right unilateral adrenal adenoma. The dosage of aldosterone/renin activity returned to 163 (normal <61). This clinical and biological picture, in particular arterial hypertension, hypokalemia, high aldosterone/renin ratio and adrenal adenoma on imaging, most likely evokes primary hyperaldosteronism of Conn's adenoma.

**Therapeutic intervention:** the patient is put on Aldactone 300 mg/day and Amlodipine 5 mg/day with correction of hypokalemia by injectable supplementation, and a potassium-rich diet discontinued after introduction of Spironlactone. After six weeks, the patient had an adrenalectomyactually.

**Follow-up and outcomes:** the evolution has been marked by a return to a premorbid state with total cleansing of psychotic symptoms.

**Patient perspective:** the patient was very satisfied with our treatment, and would like to share his experience with other patients.

**Informed consent:** the patient gave her consent for her case to be published.

## Discussion

Our patient represents one of the very few cases reported in the international literature describing an acute psychotic symptomatology inaugurating a primary hyperaldosteronism. This psychiatric picture had required hospitalization in a psychiatric department and faced with the occurrence of hypertensive peaks intra-hospital, we sent the patient for a cardiological assessment, and it is at this level that we were able to discover primary hyperaldosteronism on Conn's adenoma. The patient's journey as well as the atypical psychiatric symptomatology illustrate the interest and originality of our clinical case and justify its sharing with the psychiatric and cardiological scientific community. Cases of depression, anxiety disorders, panic attack and bipolar disorder have been identified as symptoms associated with this syndrome, however, the association between the latter and acute psychotic episodes is very rare [5]. Mood disorders are common during secreting adrenal adenomas. Major depressive disorders were observed in 50-70% of cases, anxiety disorders in 12-79% of cases, and hypomania in 3% [6]. We illustrate the complexity and interactions between adrenal adenoma and psychotic symptoms through a clinical case of a patient with portico-adrenal adenocarcinoma [7].

A close comorbidity between endocrine diseases and psychiatric symptoms has been described in the literature. So far, only a few studies have reported the prevalence of anxiety and depressive symptoms in patients with primary hyperaldosteronism [8]. The exact pathways of psychiatric comorbidities have not been fully clarified, although the renin-angiotensin-aldosterone system has gained more attention in research on anxiety and depression. Several structures could be involved in the pathophysiology of these psychopathological symptoms, among them a disturbed expression of mineralocorticoid (MR) receptors, with a modified balance between glucocorticoid receptors (GR) and (MR) that could play a role in the pathophysiology of depression and anxiety [9], this structure can be altered by the excessive secretion of aldosterone characteristic of Conn syndrome. A review of psychotic symptoms in patients with primary hyperaldosteronism by Kunzel found that while the exact pathways of psychiatric comorbidities in patients with hyperaldosteronism have not been elucidated, the renin-angiotensin-aldosterone system appears to play a role and is a target for future research in this area.

## Conclusion

Our case is a rare presentation illustrating, the association between the acute psychotic episode and the primary hyperaldosteronism. The psychiatrist should pay attention to the association between psychiatric symptoms and hypertensive peaks and think about this endocrine condition. On the other hand, more studies are needed to elucidate how high levels of aldosterone can cause or contribute to the persistence of psychotic symptoms.
